# Supplementary material for: Complex multiannual cycles of Mycoplasma pneumoniae: Persistence and the role of stochasticity
Source: Proc Natl Acad Sci U S A. 2025 Dec 22;122(52):e2509184122. doi: 10.1073/pnas.2509184122 (PMC12771566; doi:10.1073/pnas.2509184122)
Supplement: Supplementary file 1 — Appendix 01 (PDF) [file pnas.2509184122.sapp.pdf]

SI Appendix: Complex multiannual cycles of *Mycoplasma pneumoniae*: persistence and the role of stochasticity

Bjarke Frost Nielsen[a,b,c,1], **Sang Woo Park**[d,e], **Emily Howerton**[f], **Olivia Frost Lorentzen**[g], **Mogens H. Jensen**[b], **and Bryan T. Grenfell**[f]

[a]High Meadows Environmental Institute, Princeton University, Princeton, NJ, USA; [b]Niels Bohr Institute, University of Copenhagen, Copenhagen, Denmark; [c]PandemiX Center, Roskilde University, Denmark; [d]Department of Ecology and Evolution, University of Chicago, Chicago, IL, USA; [e]School of Biological Sciences, Seoul National University, Seoul, Korea; [f]Department of Ecology and Evolutionary Biology, Princeton University, Princeton, NJ, USA; [g]Canal Pointe, Princeton, NJ, USA

Bayesian fitting of 1958-1995 MpCF data As for the 2010-2025 data, we fit a seasonal SIRS model using the probabilistic programming language Stan (1, 2), with a sinusoidal seasonally forced transmisson rate $\beta(t) = \beta_0(1 + a\sin(2\pi t/\text{year} + \phi))$. We fit the modeled incidence to the variance-stabilized (square root-transformed) number of samples simultaneously positive for CA and MpCF (test data from (3)). There are no NPI multipliers in this fit, but to account for slowly changing contact rates and ascertainment patterns over the years, we included multipliers for the transmission rate $\beta(t)$ and the ρ, the constant of proportionality between measured and simulated incidence. These multipliers had relatively narrow priors centered around unity and were piecewise constant for each four years of the simulation. The priors used for the 1958-1995 fit were mostly similar to those for the 2010-2025 fit, but due to the superior data quality of the 2010-2025 data, we constrained some variables using results from that fit:

- $\beta_0 \sim N[0; 5/T]$ where $N[x; y]$ indicates a Gaussian (normal) distribution with mean x and standard deviation y.
- $\log(\rho) \sim N[-2; 0.5]$
- $S(t = 0) \sim N[0.6; 0.2]$
- $\log(x_I) \sim N[-4; 2]$ such that $I(t = 0) = x_I S(t = 0)$ (the stick-breaking method, ensuring $S(t = 0) + I(t = 0) \leq 1$)
- $\phi \sim U[0; 2\pi]$ where $U[x; y]$ indicates the uniform distribution with support on the interval $[x, y]$
- $a \sim N[0; 0.10]$
- $\beta(t)$ multipliers $\sim N[0.8; 0.3]$
- ρ multipliers $\sim N[1; 0.2]$
- $\rho x_{\text{data}} = N[x_{\text{model}}; \sigma_{\text{obs}}]$
- $\sigma_{\text{obs}} \sim N[0; 0.1]$

Here x_{data} and x_{model} refer to the measured incidence proxy and the modeled incidence, respectively. ρ is a constant of proportionality between the two. Note that for the parameters β_0, ρ, $S(t = 0)$, a, x_{data}, σ_{obs} and the $\beta(t)$ and ρ multipliers, only the part of the normal distribution with positive support is used, since the parameters in question are necessarily non-negative. The immunity rate of waning δ was fixed at the value obtained in the 2010-2025 plot.

Lyapunov exponents In Fig. S1, we show the local/instantaneous Lyapunov exponents (LLEs) $\lambda_i(t)$ of the system, as well as the cumulative means $\bar{\lambda}_i(t)$. The latter are the mean Lyapunov exponents during the first t years, i.e. $\bar{\lambda}_i(t) = \frac{1}{t}\int_0^t \lambda_i(t')\mathrm{d}t'$, the sign of which indicates whether a perturbation has undergone net growth or decay during the first t years after being applied. We show the cumulative LLEs for the system with and without seasonal forcing and noise. Local Lyapunov exponents were computed using the QR method described in (4). Conceptually, this corresponds to initially applying perturbations along the three coordinate axis (S, I, R) and then tracking how the flow (as given by the Jacobian) expands/contracts each perturbation, while taking into account that the flow also reorients the perturbation vectors.

Metapopulation simulations The metapopulation simulations of Supplementary Fig. S11 use the Doob-Gillespie algorithm as described in *Materials and Methods*, but with modified rates. There are $M > 1$ subpopulations, each with population size N_i and populations of susceptible, infected, and recovered S_i, I_i, and R_i (considered as integers). The transition rates,

Table S1. Estimated and assumed parameters from fit to 1958-1995 *Mycoplasma pneumoniae* surveillance data from Denmark.

Parameter	Description	Estimated value [95% HPD]
β_0	Mean transmission rate	0.63/wk $[0.58; 0.68]$
a	Amplitude of seasonality	0.13 $[0.11; 0.16]$
**Parameter**	**Description**	**Assumed value**
μ	Birth/death rate	$1/(75\text{yr})$
γ	Recovery rate	$1/(2.5\text{wk})$
δ	Rate of waning of immunity	$1/(8.2\text{yr})$ (from 2025 fit)

[1]To whom correspondence should be addressed. E-mail: bjarke@princeton.edu

within a given population i, are:

$$
\begin{aligned}
r[\emptyset \to S_i] &= \mu N_i, \\
r[S_i \to \emptyset] &= \mu S_i, \\
r[I_i \to \emptyset] &= \mu I_i, \\
r[R_i \to \emptyset] &= \mu R_i, \\
r[S_i \to I_i] &= \beta(t) S_i \left(c_{\text{within}} \frac{I_i}{N_i} + \frac{c_{\text{between}}}{M-1} \sum_{j \neq i} \frac{I_j}{N_j} \right) \\
r[I_i \to R_i] &= \gamma I_i, \\
r[R_i \to S_i] &= \delta R_i,
\end{aligned}
\qquad [\text{S1}]
$$

with $c_{\text{between}} = 1 - c_{\text{within}}$ the fraction of contacts that occur between two different subpopulations. Fractions such as I_i/N_i evaluate to zero if N_i (and thus also I_i) is zero.

Power spectral density of fluctuations due to demographic noise Our derivation of the power spectral density (PSD) of fluctuations in the non-seasonal SIRS model with demographic stochasticity is based directly on the methods of (5) and (6), who give a very detailed derivation in their supplement for the open SIR model. We will thus only state the principles behind the derivation, and point out the differences when applied to the SIRS model. Note that here, as in the Doob-Gillespie section, S, I, and R denote the populations of susceptible, infected, and recovered individuals, not the population fractions. Instead, we will use $\phi(t)$ and $\psi(t)$ to refer to the population fractions of susceptible and infected in the large-N limit, following (6). Since $R = N - S - I$, we leave out the population fraction of recovered from the description. In the large-N limit, the differential equation governing ϕ and ψ will take the form of Eqs. 2-3:

$$
\begin{aligned}
\frac{\mathrm{d}\phi}{\mathrm{d}t} &= \mu - \beta\phi\psi + \delta(1 - \phi - \psi) - \mu\phi, \\
\frac{\mathrm{d}\psi}{\mathrm{d}t} &= \beta\phi\psi - (\gamma + \mu)\psi.
\end{aligned}
$$

However, at finite N there will be corrections to the quantities S and I due to stochastic finite-size effects. At next to leading order in the system size expansion, one writes $S = \phi + x/\sqrt{N}$ and $I = \psi + y/\sqrt{N}$, such that x and y are the normalized finite-size fluctuations.
A set of Langevin equations for x and y can be derived (6) in the form:

$$
\begin{aligned}
\frac{\mathrm{d}x}{\mathrm{d}t} &= a_{11}x + a_{12}y + \eta_1(t), \\
\frac{\mathrm{d}y}{\mathrm{d}t} &= a_{21}x + a_{22}y + \eta_2(t),
\end{aligned}
$$

where a_{ij} are the elements of the Jacobian of the SIRS system at the (large-N) equilibrium, while η_1 and η_2 are noise terms with correlation $\langle \eta_i(t), \eta_j(t') \rangle = B_{ij}\delta(t - t')$, where $\delta(t)$ is Dirac's delta. The B_{ij} are the elements of the noise correlation matrix which, in the case of the SIRS model, are given by:

$$
\begin{aligned}
B_{11} &= \frac{2(\beta - \gamma - \mu)(\gamma + \mu)(\delta + \mu)}{\beta(\gamma + \delta + \mu)}, \\
B_{12} = B_{21} &= \frac{(\gamma + \mu - \beta)(\delta + \mu)(\gamma + 2\mu)}{\beta(\gamma + \delta + \mu)}, \\
B_{22} &= \frac{2(\beta - \gamma - \mu)(\gamma + \mu)(\delta + \mu)}{\beta(\gamma + \delta + \mu)}.
\end{aligned}
$$

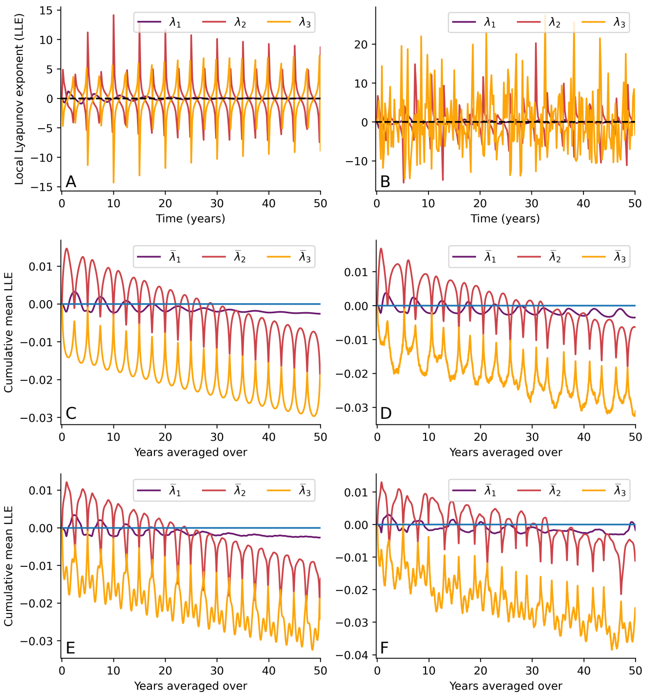

Fig. S1. Local Lyapunov exponents of the *M. pneumoniae* SIRS model A) Local/instantaneous Lyapunov exponents (LLEs) of the autonomous (unforced) system without noise ($\sigma = 0$). **B)** LLEs of the autonomous (unforced) system with noise ($\sigma = 0.12$). **C)** Cumulative mean local Lyapunov exponents $\bar{\lambda}(t) = \frac{1}{t}\int_0^t \lambda_i(t')\mathrm{d}t'$ with λ_i the i'th local Lyapunov exponent of the autonomous (unforced) system without noise ($\sigma = 0$). **D)** Cumulative mean local Lyapunov exponents $\bar{\lambda}(t)$ of the autonomous (unforced) system with noise ($\sigma = 0.12$). **E)** Same as C, but with seasonality. **F)** Same as D, but with seasonality. Evidently, the cumulative mean is similar overall, regardless of seasonal forcing.

Upon Fourier transformation of the above Langevin equation, one finds the power spectral density (PSD) of the infected class, $P_I(\omega) = \left\langle |\tilde{x}(\omega)|^2 \right\rangle$ (with $\tilde{x}(\omega)$ the Fourier transform of $x(t)$) to be given by:

$$
P_I(\omega) = \frac{\alpha_I + B_{22}\omega^2}{(\omega^2 - \Omega_0^2)^2 + \Gamma^2\omega^2}, \qquad [\text{S2}]
$$

$\Omega_0^2 = (\beta - \gamma - \mu)(\delta + \mu)$ and $\Gamma = \frac{(\beta+\delta)(\delta+\mu)}{\gamma+\delta+\mu}$ (as given in *Methods and Materials*) and $\alpha_I = a_{21}^2 B_{11} - 2a_{11}a_{21}B_{12} + a_{11}^2 B_{22}$. An analogous expression can be derived for the normalized fluctuations in the susceptible population. In the main text, for brevity, we refer to $P_I(\omega)$ just as $P(\omega)$ and let $B = B_{22}$ and $\alpha = \alpha_I$, since we are only interested in the fluctuations of the infected population.

1. Stan Development Team, Stan reference manual (2024) v2.36.0, https://mc-stan.org.
2. Stan Development Team, CmdStanPy (2024) v1.2.5, https://pypi.org/project/cmdstanpy.
3. K Lind, M Benzon, S Jensen, W Clyde, A seroepidemiological study of mycoplasma pneumoniae infections in denmark over the 50-year period 1946–1995. *Eur. J. Epidemiol.* **13**, 581–586 (1997).
4. T Okushima, Finite-time lyapunov exponents in many-dimensional dynamical systems. *Geom. Struct. Phase Space Multidimens. Chaos: Appl. to Chem. React. Dyn. Complex Syst.* **130**, 501–518 (2005).
5. D Alonso, A McKane, Extinction dynamics in mainland–island metapopulations: an n-patch stochastic model. *Bull. mathematical biology* **64**, 913–958 (2002).
6. D Alonso, AJ McKane, M Pascual, Stochastic amplification in epidemics. *J. Royal Soc. Interface* **4**, 575–582 (2007).

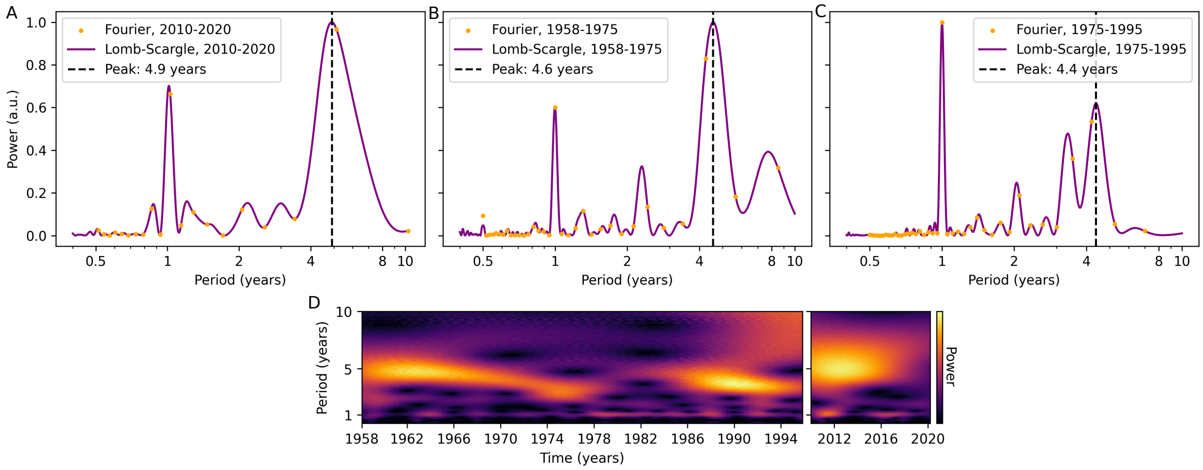

Fig. S2. Periodicity of Danish *M. pneumoniae* incidence time series. A) Fourier spectrum and Lomb-Scargle periodogram for the period January 2010 - March 2020 (prior to COVID-19 public health interventions). **B)** Spectra for the period February 1958 - January 1975. **C)** Spectra for the period January 1975 - November 1995. **D)** Wavelet transform of incidence data showing the predominantly 4-5 year period, with an absence of a clear multiannual cycle in the late 1970s and early 1980s.

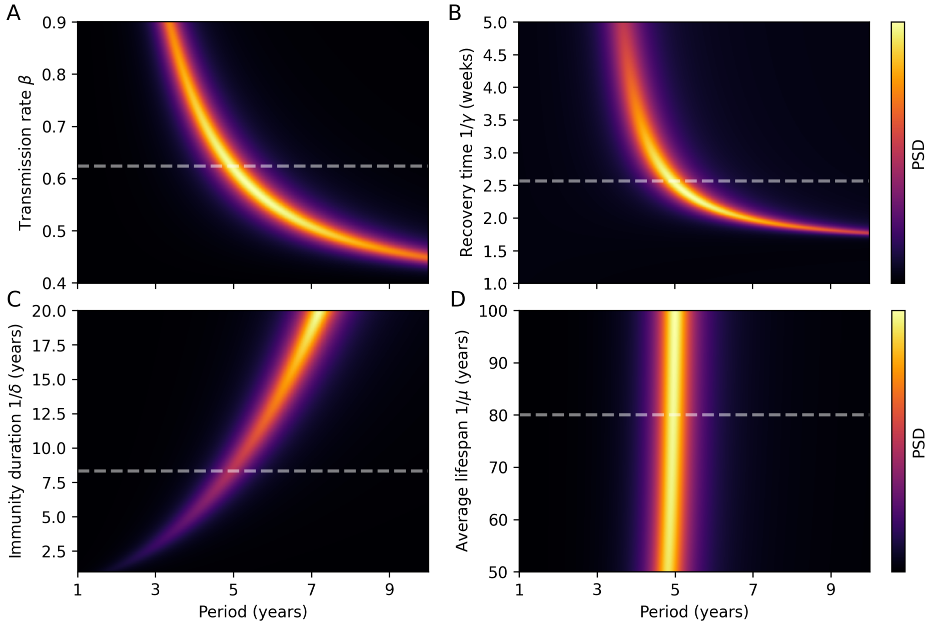

Fig. S3. Parameter dependence of the power spectral density (PSD) of fluctuations. A) Dependence on the transmission rate β. The value of the transmission rate from the 2010-2025 fit is indicated by the gray dashed line. **B)** Dependence on the recovery time (reciprocal of the recovery rate γ). **C)** Dependence on the immunity duration (reciprocal of the rate of waning δ). **D)** Dependence on the avg. lifespan (reciprocal of the birth/death rate μ). All other parameters were set to their fitted values, from the 2010-2025 deterministic model fit.

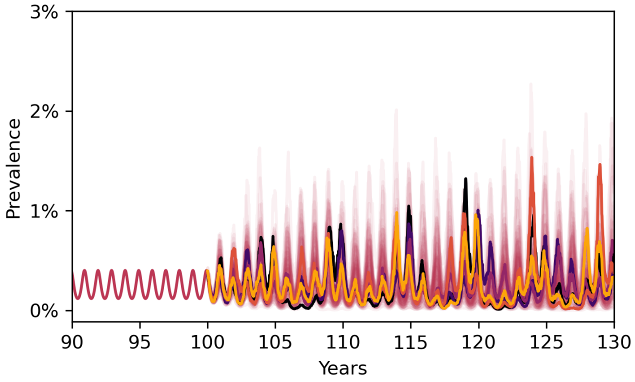

Fig. S4. Noise in transmission excites multiannual cycles starting from the annual attractor. After allowing the seasonal SIRS model to settle on the annual attractor, noise in the transmission rate $\beta(t)$ is "turned on" (noise level $\sigma = 0.15$) at $t = 100$yr. 100 realizations are shown, with five of them highlighted to clearly show the multiannual cycles which develop.

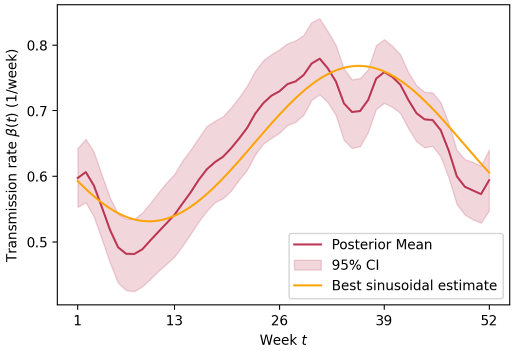

Fig. S5. Seasonally varying transmission rate. The dark red curve is the transmission rate obtained when fitting a seasonal profile. The orange curve is the transmission rate when instead imposing a sinusoidal shape, $\beta(t) = \beta_0(1 + a\sin(2\pi t/\text{year} + \phi))$, and fitting the parameters β_0, a, and ϕ.

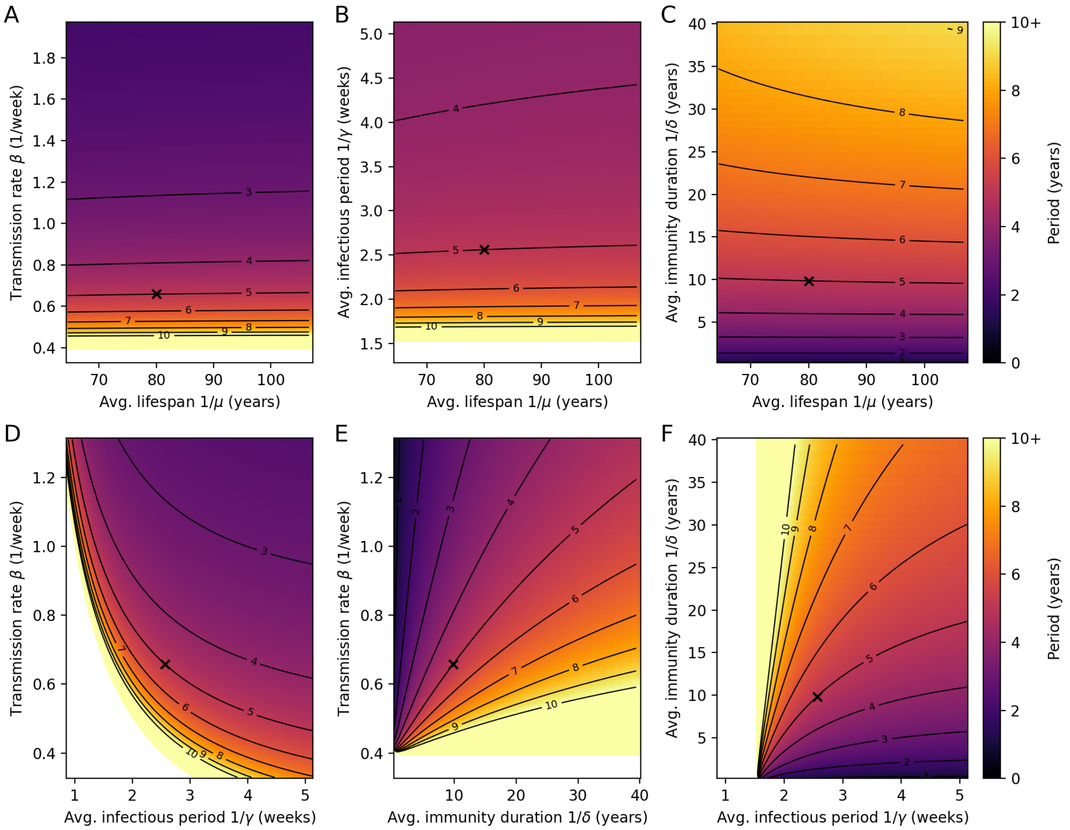

Fig. S6. Parameter dependence of the intrinsic period. Pairwise parameter dependence of the intrinsic period T, as given by Eq. 5 of the main text. In each plot, the black cross indicates the value obtained from fitting the deterministic model to the 2010-2025 data. In each plot, non-varied parameters are fixed at their fitted values. **A)** $T(\mu, \beta)$. **B)** $T(\mu, \gamma)$. **C)** $T(\mu, \delta)$. **D)** $T(\gamma, \beta)$. **E)** $T(\delta, \beta)$. **F)** $T(\gamma, \delta)$.

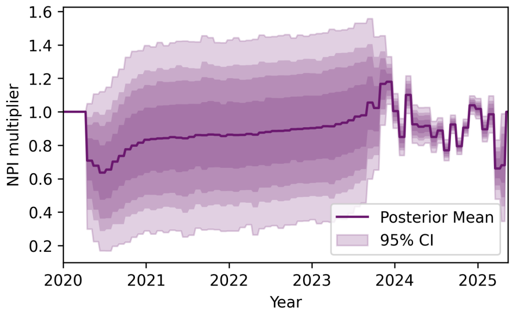

Fig. S7. NPI multipliers. Estimated transmission rate modulating factor due to interventions. Until March 2020, this multiplier was fixed at 1.0. The 95% confidence interval is shown in light purple, and the 85%, 75%, and 50% intervals are shown in successively darker shades.

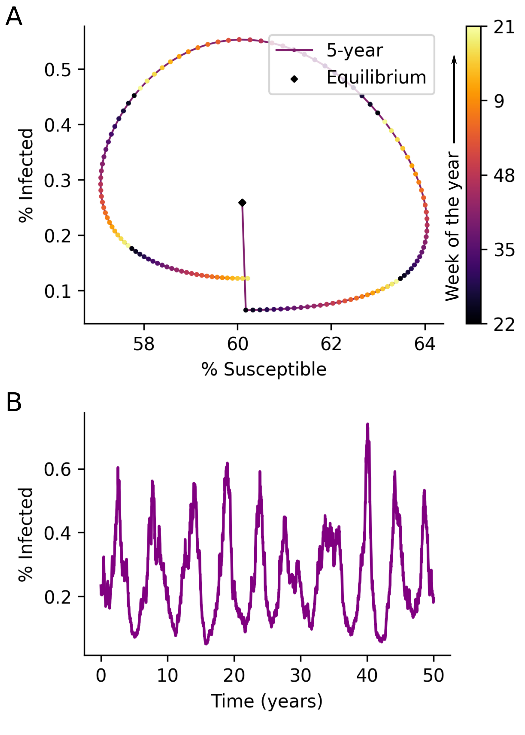

Fig. S8. Oscillations without seasonal forcing. **A)** In the non-seasonal case ($a = 0$, all other parameters as in the 2010-2025 model fit), the deterministic model tends to a (constant) endemic equilibrium (indicated by the black diamond-shaped marker). Here, the system is perturbed as in Fig. 4A, and a slowly decaying five-year cycle results. Marker color indicates time of year (as in Fig. 4A), and markers are two weeks apart for legibility. **B)** Adding environmental noise (stochasticity in β, with strength $\sigma = 0.12$) to the non-seasonal model results in sustained 5-year cycles. A transient of 50 years was discarded.

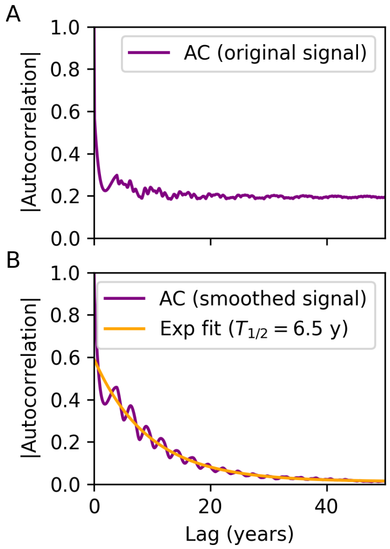

Fig. S9. Autocorrelation in the seasonal SIRS model using parameters fitted to 2010-2025 *M. pneumoniae* data. Autocorrelation at lag τ was computed according to $c_\tau = \sum_t (I(t+\tau) - \langle I \rangle)(I(t) - \langle I \rangle)/\sigma_I^2$ such that the autocorrelation at zero lag is 1 by construction ($c_0 = 1$). **A)** Absolute autocorrelation of the non-smoothed signal. **B)** Absolute autocorrelation after smoothing the $I(t)$ signal using a Savitzky-Golay filter with a window length of two years. The autocorrelation functions were similarly smoothed, for legibility.

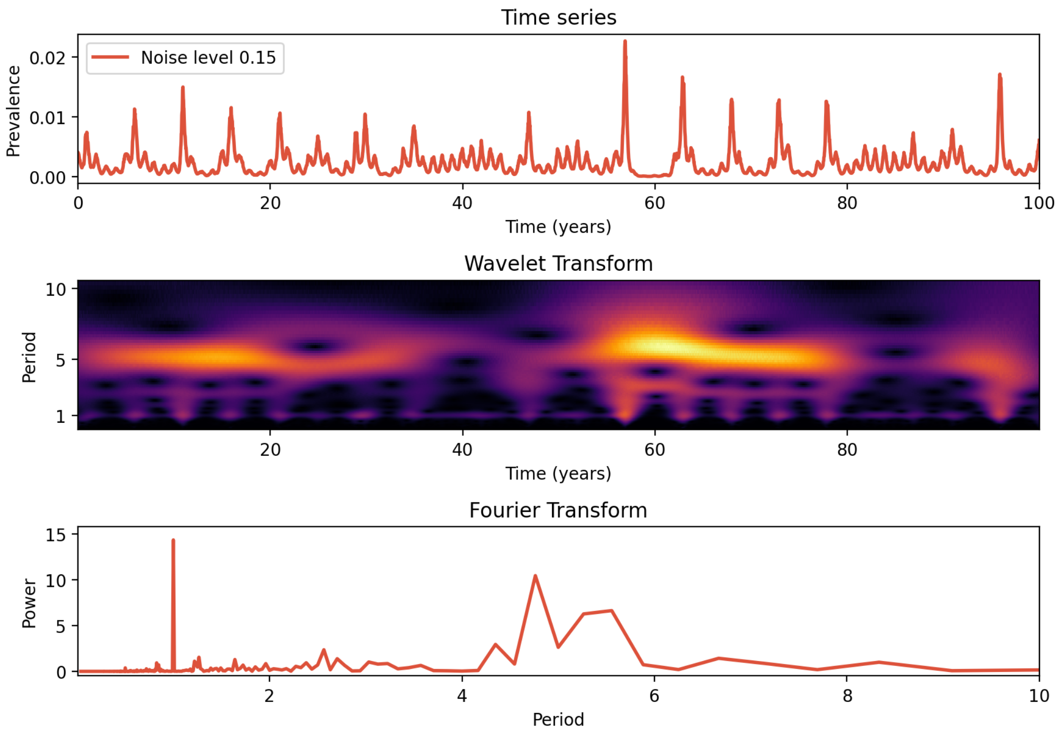

Fig. S10. The seasonal SIRS model with (environmental) noise exhibits mode-hopping. A) 100-year simulated time-series with noise level $\sigma = 0.15$. **B)** Continuous wavelet transform of the signal from panel A. **C)** Fourier transform of the entire 100-year time series. The wavelet transform confirms that the system transitions in and out of an approximately 5-year cycle, sometimes displaying primarily annual dynamics.

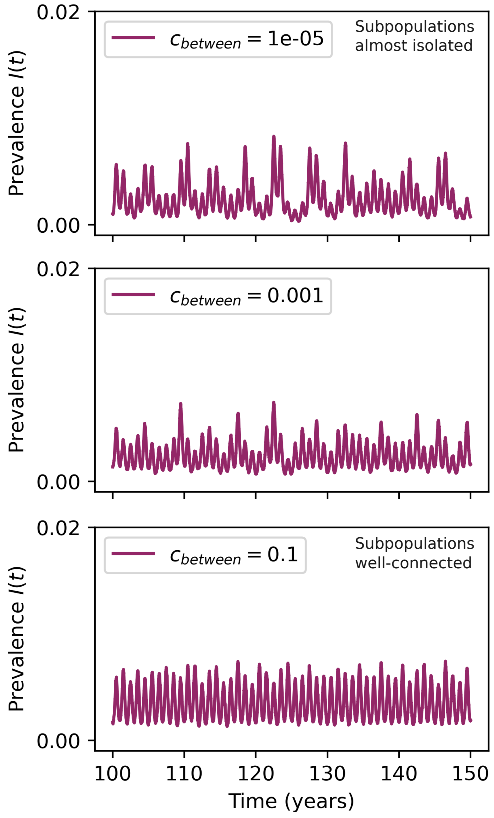

Fig. S11. Demographic noise in a metapopulation model. Doob-Gillespie simulation of the SIRS model in a population of 2.5×10^6 individuals, divided into five subpopulations of equal size, with a between-population contact fraction c_{between} and within-population contact fraction $1 - c_{\text{between}}$ (see supplement for details). The figure shows the prevalence within a single subpopulation. When interactions are predominantly local (top panel), demographic noise plays a larger role. However, when subpopulations are well-connected (bottom panel), the effects of demographic noise are washed out and multiannual cycles are not stimulated in a population of this size.
